# Electrochemical Properties of Chemically Processed SiO_*x*_ as Coating Material in Lithium-Ion Batteries with Si Anode

**DOI:** 10.1155/2014/528496

**Published:** 2014-06-22

**Authors:** Hee-June Jeong, Hyeon-Woo Yang, Kang-Seop Yun, Eul Noh, Sang-Chul Jung, Wooseung Kang, Sun-Jae Kim

**Affiliations:** ^1^Institute/Faculty of Nanotechnology & Advanced Materials Engineering, Sejong University, Seoul 143-747, Republic of Korea; ^2^Department of Environmental Engineering, Sunchon National University, Suncheon, Jeonnam 540-742, Republic of Korea; ^3^Department of Metallurgical and Materials Engineering, Inha Technical College, Incheon 402-751, Republic of Korea

## Abstract

A SiO_*x*_ coating material for Si anode in lithium-ion battery was processed by using SiCl_4_ and ethylene glycol. The produced SiO_*x*_ particles after heat treatment at 725°C for 1 h were porous and irregularly shaped with amorphous structure. Pitch carbon added to SiO_*x*_ was found to strongly affect solid electrolyte interphase stabilization and cyclic stability. When mixed with an optimal amount of 30 wt% pitch carbon, the SiO_*x*_ showed a high charge/discharge cyclic stability of about 97% for the 2nd to the 50th cycle. The initial specific capacity of the SiO_*x*_ was measured to be 1401 mAh/g. On the basis of the evaluation of the SiO_*x*_ coating material, the process utilized in this study is considered an efficient method to produce SiO_*x*_ with high performance in an economical way.

## 1. Introduction

Nowadays, portable consumer electronic products tend to be lighter and miniaturized with more functions [1–3]. With these trends, lithium-ion batteries, which are used as power source for electronic products, are required to possess high energy densities [4]. In particular, increased demands for electric vehicles and energy harvesting systems require the development of high-capacity electrodes that can be used in lithium-ion batteries. The theoretical specific capacities of the conventional cathode of LiCoO_2_ and anode of carbon are 135 and 372 mAh/g, respectively. The capacity of the cathode has been improved to about 200 mAh/g by employing new material systems [5–7]. However, the extent of capacity improvement of cathode materials was limited to less than 20% only. Thus, current situations necessitate the development of anode materials with higher specific capacity than carbon or graphite for modern electronic applications. As a way to improve the capacity of anode materials, much attention has been drawn to silicon-based materials. However, silicon-based electrodes, with high theoretical energy capacity of about 4200 mAh/g, were found to have some issues, such as low coulombic efficiency and cyclic stability.

The low coulombic efficiency and cyclic stability arising from the application of Si materials to lithium-ion batteries are caused principally by the poor electronic contact between Si particles because of large volume change during intercalation/deintercalation of Li ions. To improve the low electric conductivity and large volume expansion during lithium-ion insertion/extraction in the silicon-based anode, a number of approaches, such as the application of micron-/nanosized particles and the employment of coating with conductive additives, have been proposed and carried out [8–12]. Ryu et al. [8] reported an initial charge capacity of 3260 mAh/g and a discharge capacity of 1170 mAh/g for the micron-sized Si anode, obtaining relatively low initial coulombic efficiency of about 35%. The discharge capacity was also significantly decreased to about 200 mAh/g by the 10th cycle. When an anode sample made of nanosized Si particles was utilized [9], the relatively high initial capacity of about 2200 mAh/g was drastically reduced to around 500 mAh/g by the 10th cycle. Similar trends were observed for the anodes with Si dispersed in carbon matrix [10–12], in which the initial capacities of the anodes (about or less than 1000 mAh/g) were significantly reduced to less than 680 mAh/g by the 30th cycle.

Even though the performance has been progressively improved through various approaches, such as diffusion length adjustment, enhanced conductivity, and buffering of volume expansion, the costly and complicated processes required for the synthesis of materials could hinder the practicality of these methods. Ng et al. [13] reported the following promising results with carbon-coated Si nanoparticles: a specific capacity of 1489 mAh/g and high coulombic efficiency of above 99.5% even after 20 cycles. However, the process for the material preparation required high temperature and sophisticated procedures. This coating process may also be effective only for nanosized Si particles because of the limited mechanical strength of coated carbon films.

In this study, an economical way of processing SiO_*x*_ coating material with relatively high mechanical strength and cyclic performance was proposed; the coating material was synthesized from SiCl_4_ and ethylene glycol (EG), which are inexpensive and commercially available. The synthesized SiO_*x*_ coating material consists of nanosized Si particles uniformly dispersed in the SiO_2_ matrix. The current process was performed in the liquid state of the raw materials in the air, followed by heat treatment at a relatively low temperature, enabling the process to be scaled up economically for high volume production of SiO_*x*_ coating materials. As a feasibility study evaluating the potential of the synthesized SiO_*x*_ coating material, its electrochemical performance was systematically investigated. The synthesized SiO_*x*_ particles, mixed with an optimized amount of pitch carbon and conductive carbon blacks for cyclic stability and conductivity improvements, showed promising results. The particles showed a charge/discharge reversibility of 97% with a charging capacity of 536 mAh/g at the 50th cycle.

## 2. Experimental Procedure

An agglomerated, sponge-like, white gel powder was synthesized when EG (99.9%, Samchun Co.) was mixed with SiCl_4_ (99%, Wako Co.) solution in 1 : 1 volume ratio. The powder was heat-treated at 725°C for 1 h under a reduced atmosphere of N_2_ including 5 vol% H_2_ at 25 sccm flowing. The powder, which turned black after heat treatment, was hand-crushed by using a pestle and mortar. They were then mixed for 8 h under magnetic stirring at 80°C in the N-methyl-2-pyrrolidone (99.5% NMP, Sigma-Aldrich Co.) solution with various amounts of 10, 20, 30, 35, and 40 wt% pitch carbons as an additional carbon source. Most of the NMP solution was evaporated during the stirring. A little amount of NMP remained in the powder but was removed by placing the powder in a vacuum oven at 80°C for 24 h. For appropriate electrical conductivity of the powder as an electrode, the powder was heat-treated at 900°C for 1 h in Ar atmosphere. The heat-treated powder was characterized before and after pitch carbon coating by using scanning electron microscopy (SEM; S-4700, Hitachi Co.), Brunauer-Emmett-Teller (BET) analyzer (Nanoporosity-HQ, Mirae SI Co.), X-ray diffraction (XRD; D/MAX 2500, Rigaku Co.), and Raman spectroscopy (Invia Raman microscope, Renishaw Co.). Electrochemical characterizations for the pitch-coated SiO_*x*_ electrode with the composite powder were measured with R2032 coin-type cells, in which a metal lithium foil was used as the counterelectrode. All the electrodes in the cells were prepared by mixing 80 wt% composite powder added with various amounts of pitch carbons, 10 wt% conductive carbon blacks of Super-P (SP, TIMCAL, Super P Li) : Ketjen black (KB, Mitsubishi Chemical Co., EC-600JD) with 1 : 1 in weight ratio, 10 wt% PVDF binder, and NMP solvent to form a uniform slurry with 160 *μ*m thickness on the current collector of a copper foil by using doctor blade. Pitch carbon was mixed as a complementary way for cyclic stability improvement of the battery cell, even if the synthesized SiO_*x*_ (pitch carbon 0 wt% added) is expected to show relatively high stability. Conductive carbon blacks were added to facilitate the insertion/extraction of lithium ions during charge/discharge processes. A solution of 1 M LiPF_6_ dissolved in a mixture of ethylene carbonate and dimethyl carbonate (3 : 7 in wt ratio, Panaxetec) was used as the electrolyte. The electrodes were dried in a vacuum oven at 80°C for 24 h before being transferred into an Ar-filled glove box for cell assembly. The coin cells were charged and discharged between 0.01 and 2.5 V by applying a constant current of 50 mA/g for electrochemical characterization. By using electrochemical impedance spectroscopy (VMP3, Bio-Logic Co.), we carried out impedance analyses with Nyquist plot in the frequency ranges of 1 mHz to 1 MHz for the samples tested for various cycles.

## 3. Results and Discussion

**Figure 1:**
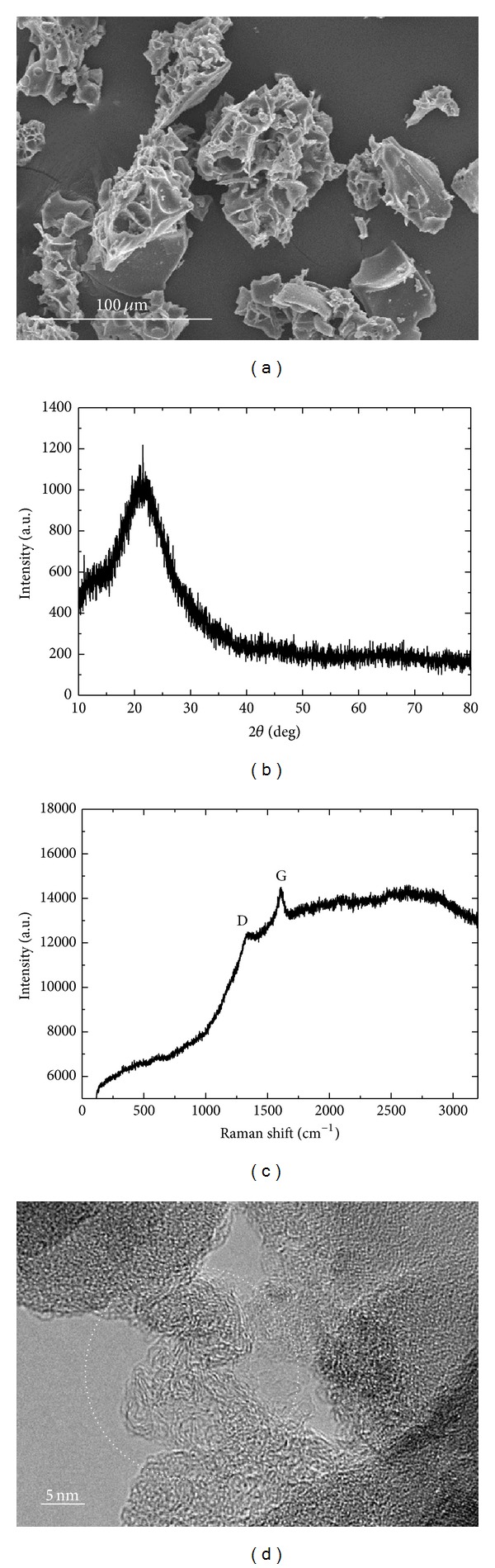
SEM photograph (a), XRD pattern (b), Raman spectrum (c), and (d) high resolution TEM micrograph for as-prepared powder, which was obtained by heat treatment of a sponge-like shaped material synthesized by chemical reaction of SiCl_4_ with EG mixed under 1 : 1 volume ratio.

Figure 1(a) shows the morphology and size of the particles observed via SEM. The materials underwent heat treatment synthesized by the chemical reaction of SiCl_4_ with EG mixed at 1 : 1 volume ratio. The chemically synthesized material with a sponge-like shape was transformed into particles by heat treatment at 725°C for 1 h under reduced atmosphere. The particles were observed to be severely agglomerated in an irregular and porous shape, and their sizes were in the less than 100 *μ*m range. The specific surface area of the powder was measured to be about 45 m^2^/g by BET, suggesting high particle porosity. The microstructural characteristics of the particles were investigated by XRD patterns and Raman spectra. The broad peaks observed from the XRD data in Figure 1(b) indicated a low degree of crystallinity or amorphous structure in the powder because no obvious crystalline peaks of Si, SiO_2_, or carbon were observed in the XRD pattern [2, 14–16]. The Raman spectrum in Figure 1(c) for the sample showed two prominent peaks of D-band at 1350 cm^−1^ and G-band at 1590 cm^−1^, representing disorder-induced and graphitic features of carbon materials, respectively [17, 18]. From the G/D ratio of 1.17, the carbon in the sample was found to have a relatively high degree of crystallinity that was not consistent with the XRD pattern. TEM micrograph taken as in Figure 1(d) confirmed a slightly crystallized microstructure of carbon (dotted circle), which was consistent with the Raman data described above. The carbon detected in the particle is considered to have originated from the starting material of EG. The content of carbon was measured by TGA analysis. The mass change of the sample was monitored as a function of temperature of up to 800°C under the air atmosphere. Assuming the sample was primarily composed of SiO_2_ and C, the carbon was measured to be approximately 14.4 wt% of the sample. Thus, the as-prepared particles consist of composites of carbon with high degree of crystallinity and SiO_*x*_ particles with amorphous or low degree of crystalline structures [4].

**Figure 2:**
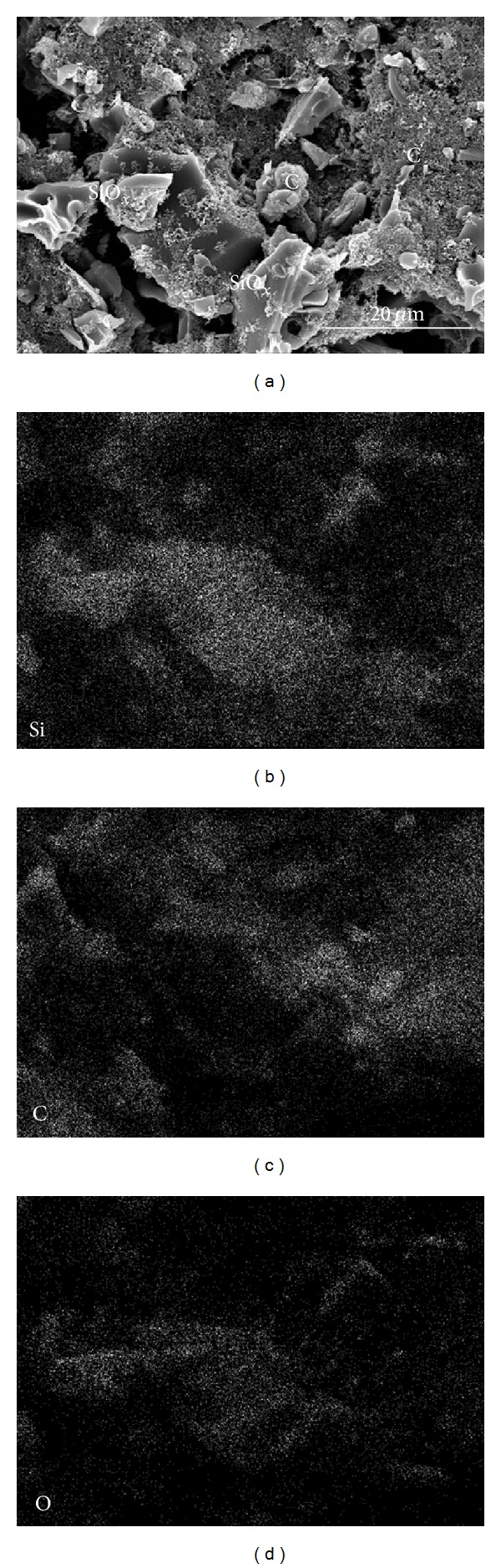
SEM photograph (a) with EDS analysis of Si (b), C (c), and O (d) elements for the surface of the anode electrode on copper foil substrate, prepared by mixing the synthesized composite of C and SiO_*x*_ particles with pitch carbon and conductive Ketjen and Super P carbon blacks.

The distribution and composition of the elements in the anode electrode were examined by using energy dispersive spectroscopy (EDS), as shown in Figures 2(a)–2(d). The constituents were shown to consist of SiO_*x*_ (detected as Si and O) with carbon homogeneously distributed. The conductive carbon blacks (20 nm to 30 nm) were distributed throughout or around the synthesized SiO particles. The XRD, Raman, and EDS data given in Figures 1 and 2 show that the synthesized particles consist of amorphous SiO_*x*_ particles as the main phase. The pitch carbon and nanosized carbon blacks homogeneously distributed on the SiO_*x*_ particles are expected to render cyclic stability and conductivity to the anode material, respectively.

**Figure 3:**
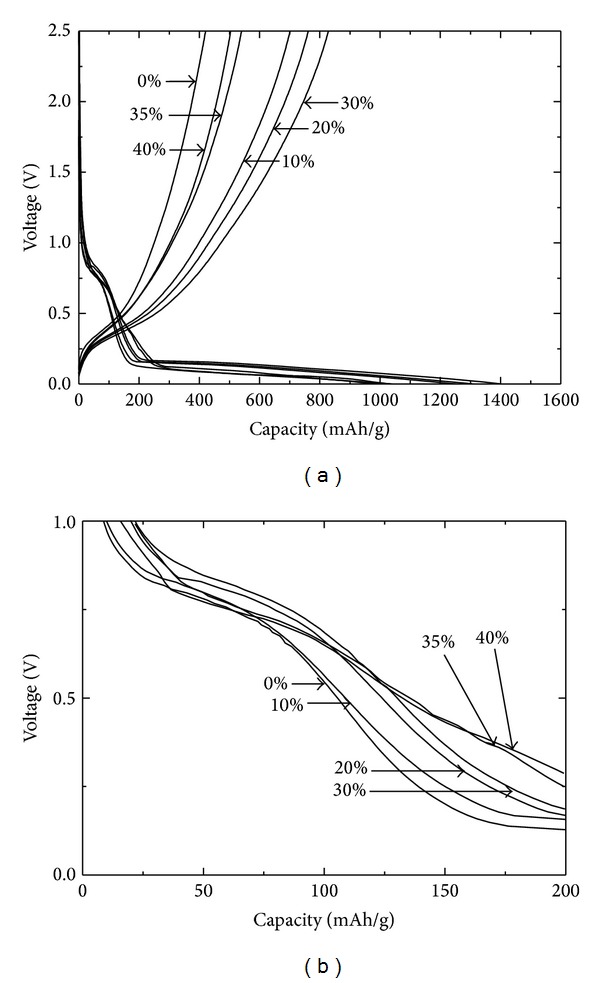
*I*-*V* characteristics during the first cycle of electrochemical charging/discharging (a) for SiO_*x*_ anode samples with different amounts of pitch carbon (0 wt% to 40 wt%) at a fixed amount of 10 wt% of conductive carbon blacks, where (b) is a magnification for the range of 0 V to 1.0 V in (a).

The effect of pitch carbon on the performance was monitored by varying its amount mixed with the as-prepared SiO_*x*_ powder. Given that the anode made with SiO_*x*_ and pitch carbon showed a low charging capacity because of the high resistivity of SiO_*x*_ (Figure 6), some conductive carbon blacks were added to the anode.  Figure 3(a) shows the initial specific capacity changes for the samples with different amounts of pitch carbon (0 wt% to 40 wt%) and a fixed amount of conductive carbon black (10 wt%) obtained through the charge/discharge test. The charging capacity of the sample without pitch carbon was measured to be 1130 mAh/g. The charging capacity was gradually increased to 1401 mAh/g when the pitch carbon increased to 30 wt%. If the amount of pitch carbon is higher than 30%, then the capacity will deteriorate. The capacity reduces when the pitch carbon is more than 30 wt% because of the ratio effect of pitch carbon with inferior capacity relative to SiO_*x*_. A similar trend of capacity variation with different amounts of pitch carbon can be observed in Figure 3(b). This trend is a magnification of Figure 3(a) for the 0 V to 1.0 V range. These data indicate the importance of optimized amounts of pitch carbon relative to SiO_*x*_ to favorably contribute to the capacity improvement by facilitating lithium-ion movements in the cell. The initial charge/discharge reversibility was also found to be affected by the amount of pitch carbon. This amount greatly improved to 59.1% when mixed with 30 wt% pitch carbon compared with 40.5% without pitch carbon. These data also show the positive effect of pitch carbon on the reversibility improvement when an optimized amount was added to the SiO_*x*_ anode.

**Figure 4:**
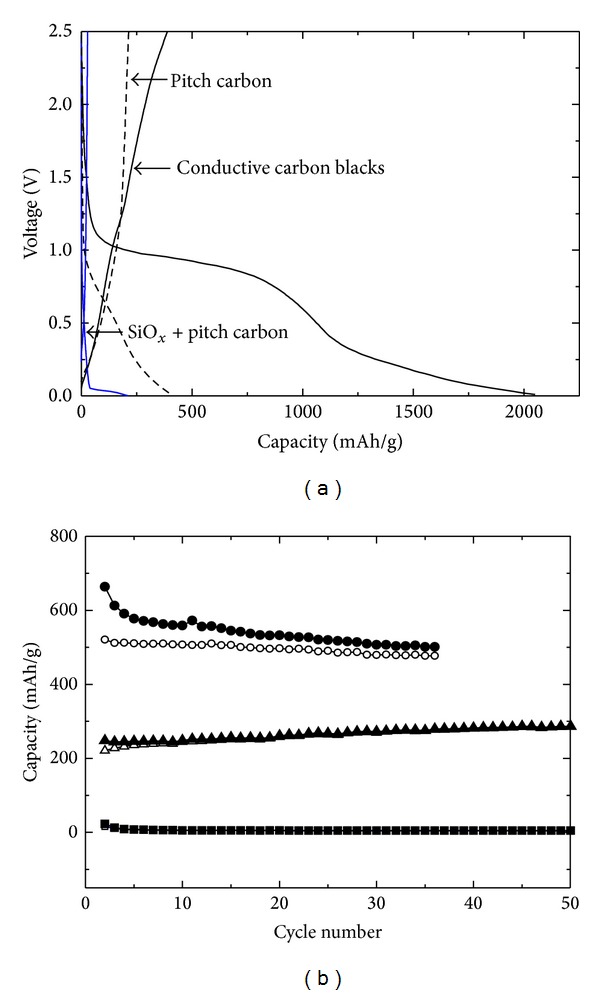
*I*-*V* characteristics during the 1st cycle of electrochemical charging/discharging (a) and the cyclic stabilities of charging (closed data)/discharging (open data) test results for the 2nd to the 50th cycle (b) for various electrodes, such as conductive carbon blacks (●, ○), pitch carbon (▲, △), and SiO_*x*_ + 30 wt% pitch carbon (■, □) samples.

In Figure 4(a), the performance of the additive materials was examined to understand individual component characteristics in terms of capacity and cyclic stability. Pitch carbon had a specific charging and discharging capacity of 426 and 210 mAh/g, respectively. The conductive carbon blacks of SP and KB (mixed with 1 : 1 wt%), which are used as conventional additives for studies on the conductive improvement of lithium-ion batteries [19], have charge/discharge capacities of 2050 and 391 mAh/g, respectively. The mixing ratio was predetermined based on our preliminary work. The conductive carbon blacks showed high capacity but poor reversibility. The SiO_*x*_ mixed with pitch carbon showed poor performance, confirming the necessity of conductive additives in the SiO_*x*_ electrode. The cyclic stabilities and charge/discharge capacities of the additives were monitored for the 2nd until the 50th cycle. The conductive carbon blacks showed a relatively poor cyclic performance in 35 cycles. Pitch carbon showed a high cyclic stability of approximately 91% at 230 mAh/g charge/discharge capacities, as exhibited in Figure 4(b). However, the SiO_*x*_ mixed with 30 wt% pitch carbon showed extremely low levels of charge/discharge capacity in the overall cyclic test. Thus, inferred from the above data, the performance of the SiO_*x*_ electrode could be enhanced by taking advantage of pitch carbon and conductive carbon blacks for cyclic stability and conductivity (or capacity) improvements.

**Figure 5:**
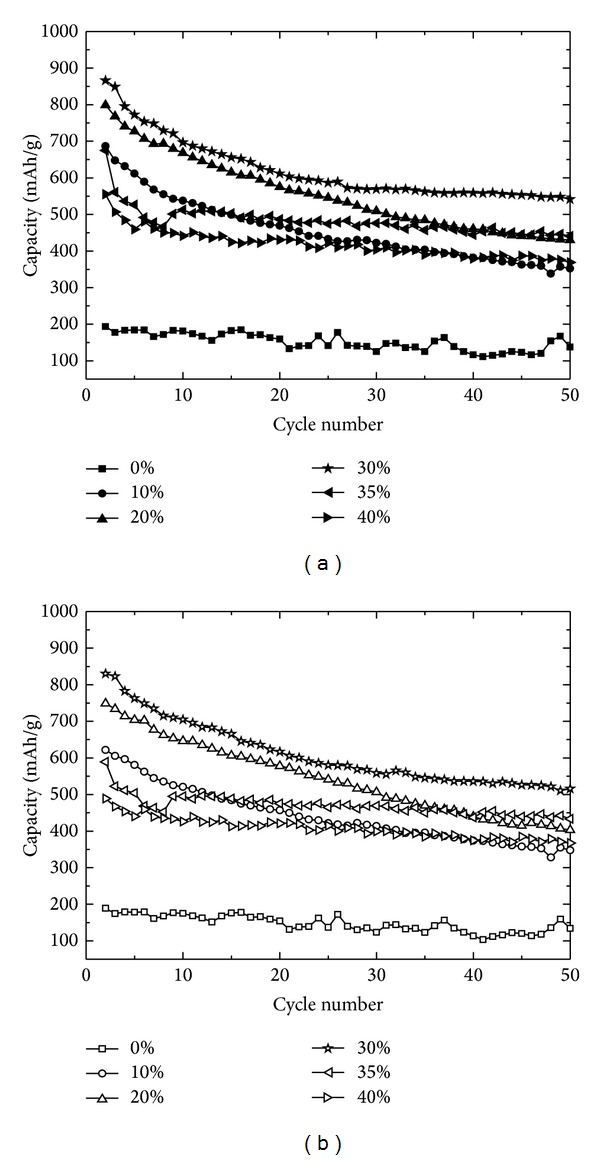
Cyclic stability test results after 2 to 50 cycles for the SiO_*x*_ anode samples with different amounts of pitch carbon (0 wt% to 40 wt%) at a fixed amount of 10 wt% of conductive carbon blacks; (a) charging state and (b) discharging state.

Figure 5 shows the specific capacity changes of the samples used in Figure 3 through the charge/discharge cyclic test of up to 50 cycles. Figures 5(a) and 5(b) show the variation of the charging and discharging capacities of the samples with the cycling test, respectively. The initial reversibility of the samples ranged from 40% to 60%, as shown in Figure 5(a). However, the reversibility significantly improved to above 97% after 30 cycles. Similarly observed in Figure 4, SiO_*x*_ mixed with the conductive carbon blacks only showed poor capacity, confirming the necessity of pitch carbon in SiO_*x*_. The capacity improved when the amount of pitch carbon increased to 30 wt%. However, a higher amount of pitch carbon will lead to deteriorated capacity. These results are consistent with the data provided in Figure 3. However, the capacity of the SiO_*x*_ electrode with more than 30 wt% pitch carbon was gradually decreased with the cycling number. On the basis of these results, 30 wt% is the threshold amount of pitch carbon needed to acquire a SiO_*x*_ electrode with high capacity and cyclic stability. To understand the cyclic stability variation with the amount of pitch carbon in the SiO_*x*_ electrode, the impedance variation through the charge/discharge cycling test was investigated by using the Nyquist plots of the samples.

**Figure 6:**
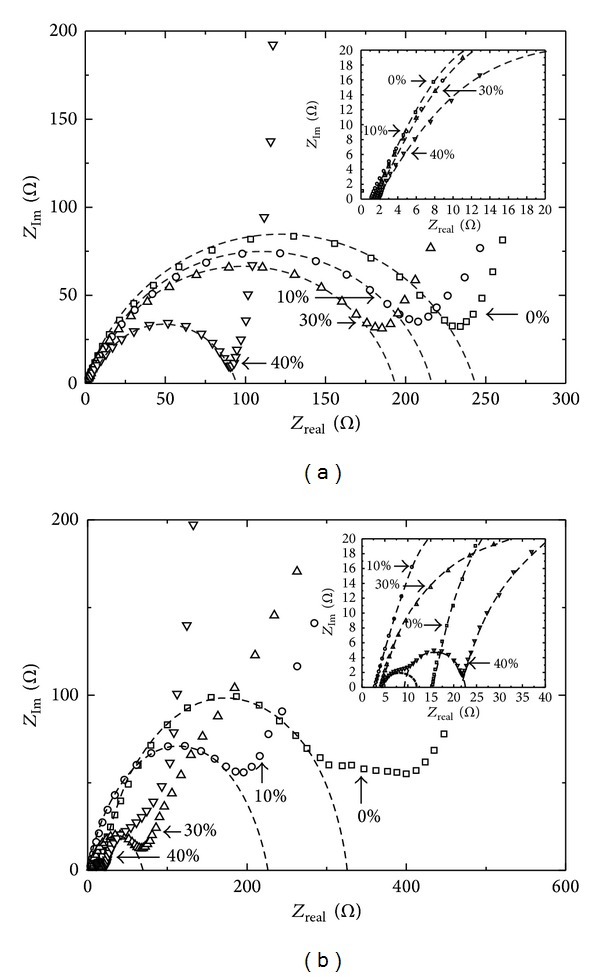
Nyquist plots for the SiO_*x*_ anode samples with different amounts of pitch carbon (0 wt% to 40 wt%) at a fixed amount of 10 wt% of conductive carbon blacks; (a) as-prepared cells and (b) after the 50th discharging state.

Figure 6(a) shows the impedance data of the samples before cycling test. In an uncharged state, the samples had resistance of 239, 212, 190, and 92 ohms with varying amounts of pitch carbon at 0, 10, 30, and 40 wt%, respectively. The initial part was magnified for a close examination and is shown in the inset. The resistance of the SiO_*x*_ electrode before cycling test decreased with the amount of pitch carbon.  Figure 6(b) shows that after 50 cycles of charge/discharge test, the resistance of the samples, except for the sample with 30 wt% pitch carbon, increased. The corresponding resistance of the samples at 0, 10, 30, and 40 wt% of pitch carbon was measured to be 306, 217, 64, and 106 ohms from the diameter of each respective semicircle. The reduced resistance in the sample with 30 wt% pitch carbon indicates that a solid electrolyte interphase (SEI) was formed and stabilized during the cycling test by electrolyte decomposition [15, 20, 21], which facilitated the charge transfer during charge/discharge processes [22]. These data are consistent with the stabilized capacities and significantly improved reversibility of the samples with cycling number, as observed in Figure 5. The newly formed semicircles at high frequencies for samples with more than 30 wt% pitch carbon also confirmed the formation and contribution of SEI to the reduced resistance of the charge flow [22].

## 4. Conclusions

SiO_*x*_ particles were synthesized as coating materials for the Si anode of lithium-ion batteries through a solution-based process by using SiCl_4_ and EG. These particles were observed to be porous and irregularly shaped with the sizes of less than 100 *μ*m. The EDS, XRD, and Raman spectra results show that the composition and microstructure of the synthesized particles consist of SiO_*x*_ (detected as Si and O) with amorphous structure. SiO_*x*_ powder was mixed with pitch carbon and conductive carbon blacks to improve cyclic stability and conductivity. The initial specific capacity was measured to be about 1401 mAh/g. The amount of pitch carbon was found to strongly affect the cyclic stability. A charge/discharge reversibility of about 97% was measured for the 2nd to the 50th cycle when an optimized amount of pitch carbon was used. Based on the impedance measurements by Nyquist plots, we determined that high reversibility and cyclic stability are caused by the stabilized SEI during cycling tests. Therefore, this study suggests that SiO_*x*_ is a promising coating material with high performance and could be easily scaled up for high volume production through an economical and efficient way of solution-based process. The electrochemical characterization of Si anode material, coated with high performance SiO_*x*_, will be carried out and reported in future studies.
